# Prenylated flavonoids from *Sophora flavescens* inhibit mushroom tyrosinase activity and modulate melanogenesis in murine melanoma cells and zebrafish

**DOI:** 10.3389/fphar.2024.1422310

**Published:** 2024-07-10

**Authors:** Fenling Fan, Lanqing Chen, Caihong Chen, Song Ang, Justin Gutkowski, Navindra P. Seeram, Hang Ma, Dongli Li

**Affiliations:** ^1^ School of Pharmacy and Food Engineering, Wuyi University, Jiangmen, Guangdong, China; ^2^ Bioactive Botanical Research Laboratory, Biomedical and Pharmaceutical Sciences, College of Pharmacy, University of Rhode Island, Kingston, RI, United States; ^3^ Guangdong Provincial Key Laboratory of Large Animal Models for Biomedicine, Wuyi University, Jiangmen, Guangdong, China

**Keywords:** *Sophora flavescens*, prenylated flavonoid, isoanhydroicaritin, kurarinone, tyrosinase, melanogenesis, zebrafish

## Abstract

**Background:** *Sophora flavescens*, a traditional Chinese medicine for treating conditions associated with abnormal skin pigmentation, contains flavonoids with inhibitory effects on tyrosinase. However, their mechanisms of action and their modulatory effects on melanogenesis remain unclear.

**Methods:** Herein, a group of prenylated flavonoids was identified from *S. flavescens* extracts and their inhibitory activities on mushroom tyrosinase were evaluated. The anti-melanogenesis effects of these prenylated flavonoids were investigated in cellular (with murine melanoma cells) and animal (with zebrafish) models.

**Results:** Prenylated flavonoids including isoanhydroicaritin (IAI), kurarinone (KR), and sophoraflavanone G (SG) were the major active constituents in *S. flavescens* extracts with anti-tyrosinase activity (IC_50_ = 0.7, 7.1, and 6.7 μM, respectively). Enzyme kinetic assays showed that IAI, KR, and SG had a mixed type of tyrosinase inhibition, supported by data from computational docking. Notably, KR at concentrations of 5 and 10 μM enhanced intracellular tyrosinase activity and stimulated melanin production in B16F10 cells, whereas SG and IAI did not exhibit significant activity. Further studies with the zebrafish model showed that IAI (80 and 160 μM) inhibited melanin biosynthesis by about 30.0% while KR (20 μM) stimulated melanogenesis by 36.9%. Furthermore, a zebrafish depigmentation model supported the anti-melanogenesis effect of IAI (80 and 160 μM) by 33.0% and 34.4%, respectively.

**Conclusion:** In summary, IAI was identified as a tyrosinase inhibitor with an anti-melanogenic effect and KR was an enhancer for melanin production in B16F10 cells and zebrafish. Findings from the current study suggest that IAI and KR from *S. flavescens* may exert contrasting effects in the modulation of melanin production, providing important insights into the development of *S. flavescens* as a cosmeceutical or medicinal ingredient.

## 1 Introduction

Melanins are biological pigments responsible for the skin, hair, and eye color generated by specialized epidermal cells (Mort et al., 2015; Karunarathne et al., 2019). Under normal physiological conditions, melanins confer protective effects against ultraviolet (UV) radiation (Jang et al., 2020; Rai et al., 2020). Defective melanin production is associated with skin hypopigmentation, which increases the risk of various disorders including skin cancers (Ahmed et al., 2021). However, excessive or frequent UV exposure can result in abnormal melanin production, leading to hyperpigmentation conditions such as melasma and age spots (Grimes et al., 2006; Choi et al., 2022). The production of melanin is a complex biological process. Tyrosinase (EC 1.14.18.1) is the main rate-limiting enzyme in biosynthesis of melanin. It catalyzes the conversion of tyrosine to dihydroxyphenylalanine (DOPA) and further oxidizes DOPA to dopaquinone. Subsequent steps involve additional enzymes, such as tyrosinase-related protein 1 (TRP-1) and dopachrome tautomerase (DCT), to produce eumelanin (Del Marmol and Beermann, 1999). Melanin production can be induced by *α*-melanocyte-stimulating hormone (*α*-MSH) and 3-isobutyl-1-methylxanthine (IBMX) (Park et al., 2011; Han et al., 2020). The activity of tyrosinase is stimulated by *α*-MSH and IBMX via the cyclic adenosine phosphate (cAMP) pathway (Kim et al., 2018). *α*-MSH binds to the melanocortin-1 receptor (MC1R) on the cell surface, activates the protein kinase A (PKA) pathway, and phosphorylates CREB transcription factors, which collectively induce the expression of microphthalmia-associated transcription factor (MITF) (D’Mello et al., 2016). MITF can regulate the expression of TRP1 and DCT by binding to the M-box of the distal tyrosinase element (TDEs) (Yasumoto et al., 1994; Yasumoto et al., 1997).

Numerous natural product-based tyrosinase inhibitors (e.g., arbutin and kojic acid) have been widely used for anti-melanogenesis (Slominski et al., 2004; Pillaiyar et al., 2017). However, several anti-melanogenic agents are reported to cause side effects such as albinism (Niu and Aisa, 2017). Natural products with the opposite effect, i.e., compounds that can enhance tyrosinase activity, may restore pigmentation as a management for pathological conditions such as vitiligo (Heriniaina et al., 2018). Thus, compounds that can modulate the production of melanins by either inhibiting tyrosinase activity or increasing melanogenesis are of interest to the biomedical and cosmetic industries (Lajis and Ariff, 2019). *Sophora flavescens* Aiton (Kushen in Chinese), belonging to the Fabaceae family, has been used in traditional Chinese medicine (TCM) for the treatment of dysentery, hematochezia, jaundice, eczema, and ulcers (He et al., 2015; Kim et al., 2018). Notably, published studies support that *S. flavescens* extracts can exert various modulatory effects on melanin-related skin conditions including hyperpigmentation (Shin et al., 2013) and vitiligo (Sun and Bao, 2008; Cai et al., 2011; Zhang and Zhang, 2020). The phytochemical investigations reported that the main active metabolites of *S. flavescens* are alkaloids and flavonoids (He et al., 2015). Some of these flavonoids are tyrosinase inhibitors (Kim et al., 2003; Son et al., 2003; Hyun et al., 2008; Kim et al., 2018). For instance, prenylated flavonoids including sophoraflavanone G (SG), kuraridin, and kurarinone (KR) from *S. flavescens* exert a strong inhibitory effect on tyrosinase activity (Kim et al., 2003). However, the tyrosinase inhibition mechanism of these prenylated flavonoids remains unclear. Moreover, the modulatory effects of *S. flavescens* prenylated flavonoids on melanogenesis in cells- or animal-based models are unknown. As a continuous effort to study the bioactive constituents of medicinal plants from the Lingnan regions in China (Li et al., 2015; Zhou et al., 2019; Xu et al., 2021; Zhang et al., 2021), we aimed to evaluate the modulatory effects of *S. flavescens* extract and its active constituents on tyrosinase activity and melanogenesis in the current study. Herein, we evaluated the inhibitory effect of *S. flavescens* extract and four prenylated flavonoids on mushroom tyrosinase *in vitro*, and the anti-tyrosinase and anti-melanogenesis effects of these flavonoids using cellular (murine melanoma cells) model. In addition, prenylated flavonoids were further evaluated for their effects on melanogenesis with a zebrafish model.

## 2 Materials and methods

### 2.1 Materials

Tyrosinase, 3,4-dihydroxy-L-phenylalanine (L-DOPA), 8-methoxypsoralen (8-MOP) (purity 98%), glabridin (purity 98%), *α*-MSH, 1-phenyl-2-thiourea (PTU), and 3-(4,5-dimethylthiazol-2-yl)-2,5-diphenyltetrazolium bromide (MTT) were purchased from Sigma-Aldrich (St. Louis, MO, United States). Kushenol I (KI) (purity>98%), isoanhydroicaritin (IAI, purity > 98%), KR (purity > 98%), and SG (purity > 98%) were purchased from Shanghai Yuanye Bio-Technology Co., Ltd. Dulbecco’s modified Eagle’s medium (DMEM), fetal bovine serum (FBS), penicillin/streptomycin (P/S) and bicinchoninic acid assay (BCA) kit were purchased from Thermo Fisher Scientific (Waltham, MA, United States). TYR, TRP-1, MITF, DCT, and *β*-actin primary antibodies were acquired from Affinity Biosciences (Woburn, MA, United States). The anti-mouse IgG and anti-rabbit IgG secondary antibodies were bought from Cell Signaling Technology (Beverly, MA, United States). The roots of *S. flavescens* from Shanxi Province were purchased from Anguo Kangde Ruiqi Trading Co., LTD.

### 2.2 Preparation of extracts and principal component analysis

Dried roots of *S. flavescens* were powdered and weighed in five portions (5 g each). Ethyl acetate, dichloromethane, 70% methanol, 70% ethanol, and dichloromethane/methanol (1:1; v/v) were used for their respective extractions. The extracts were filtered after being sonicated for 30 min at 40 kHz (500 W, 40°C) and concentrated under reduced pressure, then weighed at room temperature for use. All samples were dissolved with methanol and filtered through a membrane filter (0.22 μm). The contents of major flavonoids in them were determined by a chromatographic method performed on a waters ACQUITY UPLC H-class PLUS system coupled with a DAD detector. The separation of flavonoids was achieved on an ACQUITY-UPLC-BEH-C18 column (100 mm × 2.1 mm; 1.7 μm) at a flow rate of 0.3 mL/min with detection wavelengths of 280 nm and 295 nm. The gradient elution system consisting of solvent A (water) and solvent B (methanol) was set as follows: 0–2 min, 50%–65% B; 2–4 min, 65%–70% B; 4–6 min, 70%–75% B; 6–8 min, 75%–80% B; 8–10 min, 80% B; 10–12 min, 80%–90% B; 12–14 min, 90%–100% B; 14–18 min, 100% B; 18–20 min, 100%–50% B. The injection volume for each analysis was 2 μL and the column temperature was kept at 35°C. The peak area corresponding to different concentrations of each standard was measured and recorded. Standard curves were constructed with the concentration as the X-axis and the peak area response value as the Y-axis. The content of each compound was determined by substituting the standard curve into the test sample.

### 2.3 Cell line and cell culture

B16F10 cells were purchased from Procell Life Science and Technology Co., Ltd. (Wuhan, China). The cells were cultured in DMEM supplemented with 10% FBS and 1% penicillin and streptomycin at 37°C under an atmosphere of 5% CO_2_. To control the pH value of the culture medium to be suitable for cell growth, two equal volumes of DMEM with 3.7 g/L NaHCO_3_ or 1.5 g/L NaHCO_3_ were mixed for use.

### 2.4 Cell viability assay

B16F10 cells were seeded into 96-well plates at the density of 5 × 10^4^ cells/mL, and incubated at 37°C for 24 h. Then, the cells were treated with different concentrations of test samples for 48 h. After incubation, 20 μL MTT (5 mg/mL) was added to each well, and incubated for another 4 h at 37°C. The reaction was stopped by adding 150 μL DMSO per well. The optical density at 490 nm was measured using a microplate reader. Cell viability was calculated as the percentage of viable cells relative to the control group.

### 2.5 *In vitro* tyrosinase inhibition assay

Tyrosinase inhibition assay was measured by using L-tyrosine as a substrate according to a previously described method with minor modifications (Kim et al., 2018). Briefly, a mixture of phosphate buffer (130 μL, 50 mM, pH 6.8), tyrosinase solution (10 μL, 666.67 U/mL) and different concentrations of test samples (10 μL) was incubated in 96-well plates for 5 min. After adding substrate L-tyrosine solution (50 μL, 2 mM) to each well and incubating at 37°C for another 20 min, the absorbance was measured at the wavelength of 475 nm using a microplate reader. The inhibitory rate was calculated using the following formula:

$$\text{Inhibitory rate }\left(\%\right)=\left(1-\frac{A-B}{C-D}\right)\times 100\%$$

where A is the absorbance of the sample group, B is the absorbance of the background group, C is the absorbance of the control group, and D is the absorbance of the blank group. The test sample concentration that inhibits 50% of tyrosinase activity (IC_50_) was calculated. All tests were performed in triplicate.

### 2.6 Tyrosinase inhibition kinetic analysis

Based on IC_50_ values, test samples that showed better inhibitory activity were selected for kinetic analysis. The enzyme reaction kinetics of these compounds were measured by constructing Lineweaver-Burk plots of inverse velocities (1/[V]), contrary to the inverse of substrate concentration (1/[S]) (Yoshino and Murakami, 2009; Butt et al., 2019). Preincubations and measurement times were performed using the same protocol as described above. The substrate tyrosine concentration was kept between 0.5 and 8 mM in all kinetic studies, test sample concentrations were set as follows: glabridin (0–0.625 µM), KR (0–5 µM), SG (0–10 µM), IAI (0–2 µM). The values of the kinetic constants were calculated using Lineweaver-Burk plot analysis, and the maximum velocity (V_max_) and Michaelis constant (K_m_) were also calculated by the Lineweaver-Burk plots with the various concentrations of substrate.

### 2.7 Molecular docking

To anticipate the ligand-receptor interactions, molecular docking experiments were conducted using AutoDock Tools software. The three-dimensional structures of compounds were downloaded from https://pubchem.ncbi.nlm.nih.gov/ in SDF format, which was converted to PDB format using the Discovery Studio 4.5 software (Accelrys, San Diego, CA). A crystal structure of tyrosinase (PDB ID: 2Y9X, resolution: 2.78Å) was retrieved in PDB format from the RCSB protein data bank (https://www.rcsb.org/). Water molecules, ions, and other known ligands were removed. Then, the grid map was set to the dimensions of 60 × 60 × 60 with a spacing of 0.375 Å in the process of docking. The lowest energy docking conformation at the binding site for each compound was selected and further analyzed using Discovery Studio 4.5 software.

### 2.8 Measurement of tyrosinase activity in B16F10 cells

Intracellular tyrosinase activity was determined by measuring the rate of oxidation of L-DOPA to dopachrome according to a previously described method with a slight modification (Maack and Pegard, 2016). The tyrosinase inhibitor glabridin (Zhang et al., 2023) and tyrosinase activator 8-MOP (Yin et al., 2018) were both utilized as controls to ensure the accuracy of tyrosinase activity assay in living cells. B16F10 cells were cultured in 24-well plates at the density of 5 × 10^4^ cells/mL overnight and then treated with different concentrations of test samples for 48 h. After treatments, cells were washed twice with PBS, lysed with PBS containing 1% Triton X-100, and frozen at −80°C for 1 h. The lysates were then clarified by centrifugation for 20 min at 4°C, 12,000 r/min. The protein content in the supernatants was measured using the Pierce BCA protein assay kit. After protein quantification and the adjustment of the protein concentrations, 100 μL of each lysate was mixed with 100 μL of 4 mM L-DOPA in a 96-well plate, and then incubated at 37°C for 1 h, the optical density at 475 nm was measured using a microplate reader. Tyrosinase activity was expressed as a percentage of the control. All experiments were carried out at least three times.

### 2.9 Measurement of melanin content in B16F10 cells

Measurement of melanin content was carried out according to a previously described method with a slight modification (Karunarathne et al., 2019). B16F10 cells were cultured in 6-well plates at the density of 2 × 10^5^ cells/mL overnight before being treated with different concentrations of test samples for 48 h. After the treatments, the medium was discarded and the cells were washed twice with PBS and were collected, then 200 μL of RIPA lysis buffer was added to each well, and left standing in an ice bath for 40 min. The lysates were then clarified by centrifugation for 20 min at 4°C, 12,000 r/min. The protein content in the supernatants was measured using the Pierce BCA protein assay kit. Then the harvested cell pellets were photographed and dissolved in 100 μL of 1 M NaOH (containing 10% DMSO) at 100°C for 1 h. Melanin content was determined by measuring absorption at 405 nm. For the accurate calculation of melanin content, absorbance values were normalized to total protein absorbance values.

### 2.10 Western blotting

Equal amounts of proteins from the preceding step were loaded and separated by using the 10% SDS-PAGE gels before being electro-transferred onto polyvinylidene fluoride membranes. The membrane was then blocked with 5% skim milk in TBST (Tris-buffered saline containing 0.1% Tween 20) at room temperature for 2 h. The blots were incubated overnight with primary antibodies against TYR, TRP-1, MITF, DCT, and *β*-actin at 4°C. After washing thrice with TBST (5 min), the membrane was incubated with the secondary antibody for 1 h at room temperature. The protein bands were detected using the ECL detection kit and imaged using a chemiluminescence gel imaging system, and the band intensities were analyzed by the Image J software.

### 2.11 Origin and maintenance of zebrafish

AB strain zebrafish were purchased from Guangdong Perfect Life Health Science and Technology Research Institute Co., LTD, and maintained at 28°C in a temperature-controlled room with a 14/10 h day/night cycle. Zebrafish embryos were produced by natural mating and raised in 2,000 mL of embryo media, which included 7.0 g NaCl, 0.4 g NaHCO_3_, 0.1 g KCl, and 0.2 g CaCl_2_ in distilled water. The day before the experiment was performed, female and male zebrafish were placed in the breeding tank at a ratio of 1:1 and embryos were collected the next morning for subsequent experiments.

### 2.12 Determination of the maximum tolerated concentration (MTC)

Zebrafish embryos that were developing normally at 24 h were chosen at random, five zebrafish embryos per well were seeded in 48 well plates, and each group was set up with three duplicates. The embryo culture medium in the 48-well plate was removed except the blank group, and 1 mL of culture medium containing different concentrations of test samples was added to treatment groups for 24 h. The vehicle control group was added to the embryo culture medium containing 0.1% of DMSO. MTC was defined as the concentration group that did not show any embryonic death after 24 h of treatments.

### 2.13 Melanogenesis in zebrafish larvae *in vivo*

To assess the effects of these compounds on melanogenesis *in vivo*, the total melanin content of whole zebrafish extracts was measured according to a previously described method with minor modification (Karunarathne et al., 2019; Ding et al., 2021). Briefly, zebrafish larvae of 24 h post-fertilized (hpf) were seeded in 24-well plates with ten embryos per well, each group was set up with four replicates and pretreated with 200 μM PTU for 24 h. Then, the culture medium was replaced with different concentrations of test samples and treated for another 48 h. 8-MOP (50 μM) was used as a positive control, blank controls were not treated and were cultured normally. In addition, to further verify the whitening effect of the compounds, ten zebrafish larvae (24 hpf) per well were seeded in a 24-well plate and cultured for another 24 h using fresh culture media, and each group was set up with four duplicates. Then, the culture medium was replaced with different concentrations of test samples, and the zebrafish larvae were incubated for another 48 h. 8-MOP (50 μM) and arbutin (100 mM) were used as positive controls. Spontaneous melanin content was measured from zebrafish larvae at 96 hpf. The fish embryos were fixed upright with sodium carboxymethylcellulose fixative. Five fish embryos from each experiment were randomly selected for photography under a stereomicroscope. The densitometric analysis was performed using Image J software.

### 2.14 Statistical analyses

All data are presented as the mean ± SD of at least three independent experiments. Student t-tests were used to compare the two groups and one-way analysis of variance (ANOVA) was used to determine any statistical differences between multiple groups. When the *p*-value was less than 0.05, the difference between groups was considered statistically significant.

## 3 Results

### 3.1 Tyrosinase inhibition activity and chemical components of *Sophora flavescens* extracts

**FIGURE 1:**
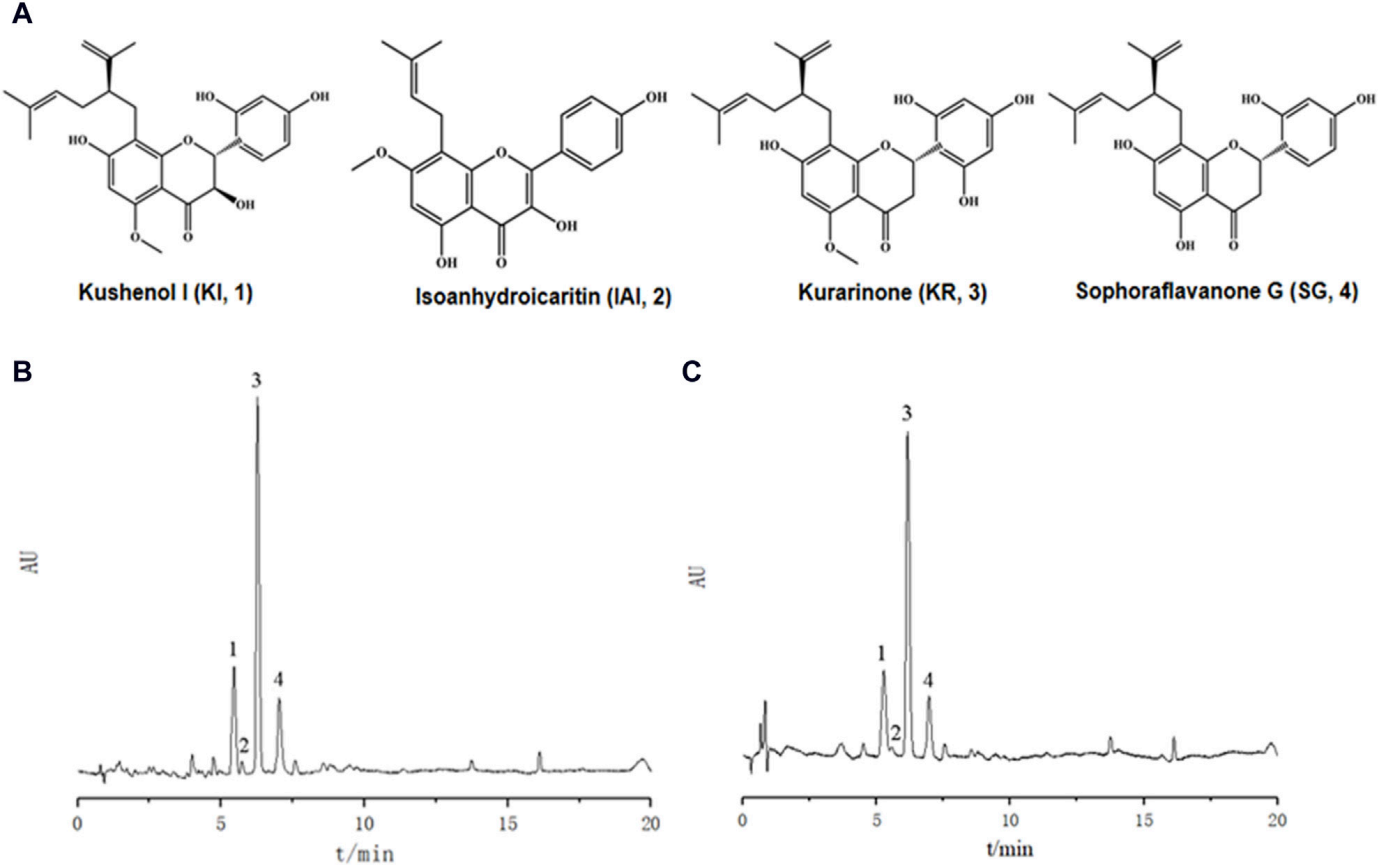
UPLC chromatograms of *Sophora flavescens* extract. **(A)** Chemical structures of the four major chemical components. **(B)** SF-EA, **(C)** SF-DM. Peak 1: KI, peak 2: IAI, peak 3: KR, peak 4: SG.

Ultrasonic-assisted extraction with five solvents yielded a 70% aqueous ethanol extract (1,518.7 mg), a 70% aqueous methanol extract (1,377.7 mg), an ethyl acetate extract (92.5 mg), a dichloromethane extract (81.0 mg) and a dichloromethane/methanol (1:1; v/v) extract (806.3 mg), respectively. Then, the inhibitory activity of each extract against tyrosinase was evaluated separately (Table 1). The extracts of ethyl-acetate (SF-EA) and dichloromethane (SF-DM) showed stronger inhibitory activity with IC_50_ values of 0.7 μg/mL and 0.4 μg/mL, respectively. Therefore, SF-EA and SF-DM extracts were selected for further qualitative and quantitative analyses of their main chemical components by UPLC (Figure 1). The peaks were assigned by comparing their retention times (t_R_) with that of each reference compound. Peaks 1–4 were determined as prenylated flavonoids KI (t_R_ = 5.3 min), IAI (t_R_ = 5.8 min), KR (t_R_ = 6.2 min), and SG (t_R_ = 7.0 min), respectively, and their content in SF-EA and SF-DM extracts were shown in Tables 2, 3.

**TABLE 1:** Yield and tyrosinase inhibitory activity of *Sophora flavescens* extract.

Solvent	Weight of extract (mg)	Rate of yield (%)	IC_50_ (μg/mL)
70% ethanol	1518.7	30.4	6.9
70% methanol	1377.7	27.6	90.0
ethylacetate	92.5	1.9	0.7
dichloromethane	81.0	1.6	0.4
dichloromethane:methanol (1:1)	806.3	16.1	5.0

**TABLE 2:** Contents of the four major components in SF-EA extract.

References	Regression equation	r^2^	Linearity range μg/mL	Content/%
KI (1)	y = 9130.2x+399.6	0.9990	1.5–59.0	2.3
IAI (2)	y = 903.8x-699.6	0.9999	1.0–8.0	3.0
KR (3)	y = 15437.0x-8480.5	0.9992	1.1–56.0	6.4
SG (4)	y = 9829.4x-6972.1	0.9994	1.2–62.0	2.8

**TABLE 3:** Contents of the four main components in SF-DM extract.

References	Regression equation	r^2^	Linearity range μg/mL	Content/%
KI (1)	y = 9130.2x+399.6	0.9990	1.5–59.0	2.1
IAI (2)	y = 903.8x-699.6	0.9999	1.0–8.0	2.8
KR (3)	y = 15437.0x-8480.5	0.9992	1.1–56.0	4.7
SG (4)	y = 9829.4x-6972.1	0.9994	1.2–62.0	2.1

In addition, we purchased the standard of the four main compounds and measured the tyrosinase activity. IAI and the positive control glabridin (a prenylated flavonoids from licorice) (Yoshioka et al., 2020) had the strongest inhibitory effect on tyrosinase with an IC_50_ value of 0.7 and 0.1 μM, respectively. KR and SG also exhibited inhibition against tyrosinase with an IC_50_ value of 7.1 and 6.7 μM, respectively. The activities of these three compounds were much higher than the other positive control kojic acid (IC_50_ = 25.9 μM), whilst KI showed weaker inhibitory activity with the IC_50_ value >80 μM (Table 4).

**TABLE 4:** Tyrosinase inhibitory activity of the four major chemical components.

Compound	IC_50_ values (μM)
KI	>80
IAI	0.7
KR	7.1
SG	6.7
Glabridin	0.1
Kojic acid	25.9

### 3.2 Kinetic analysis of tyrosinase inhibition

**FIGURE 2:**
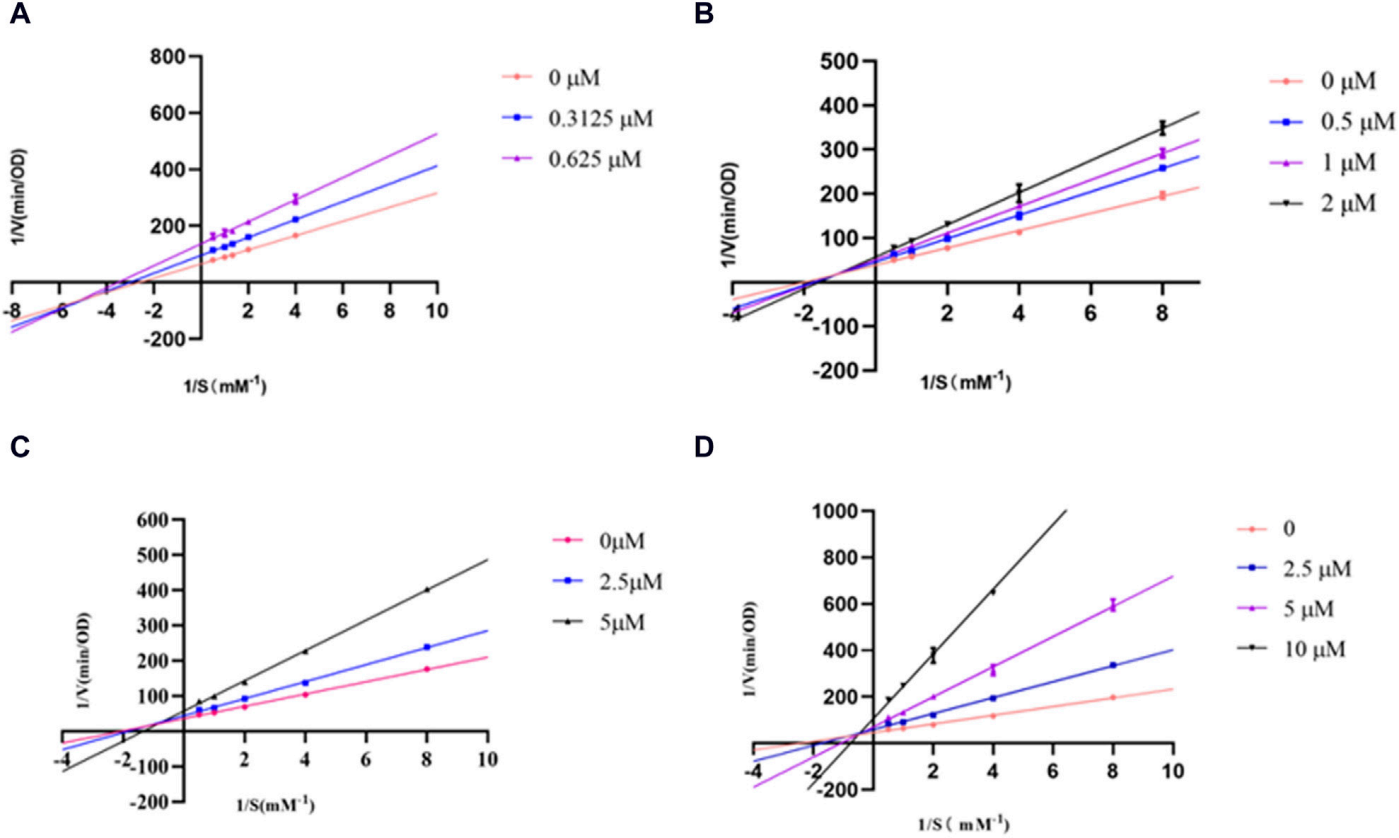
Lineweaver-Burk plots for inhibition of tyrosinase in the presence of compounds: **(A)** Glabridin, **(B)** IAI, **(C)** KR, **(D)** SG.

Next, the inhibitory mechanisms of the three most active compounds were studied by kinetic assays using L-tyrosine as a substrate. Figure 2 showed the Lineweaver-Burk double reciprocal plots (1/[V] vs. 1/[S]) for glabridin and the prenylated flavonoids. The intersection of the curves was outside the X and Y-axes, suggesting that the inhibition type of IAI, KR, and SG was a mixed type. This is characterized by the intersection of the double reciprocal lines at different concentrations above the X-axis. As the concentration increased, there was a gradual reduction in V_max_ and an increase in K_m_, resulting in a steeper slope. Table 5 showed the relevant enzyme kinetic parameters, which suggested that IAI, KR, and SG may bind to the enzyme and the substrate-enzyme complexes.

**TABLE 5:** Kinetic analysis of active compounds on tyrosinase.

Concentration (µM)	Intercept	Slope	K_m_	V_max_	Relevancy
(Glabridin) 0	65.42	25.90	0.396	0.015	0.9978
(Glabridin) 0.3125	95.97	31.66	0.330	0.010	0.9977
(Glabridin) 0.625	136.9	38.95	0.285	0.007	0.9916
(IAI) 0	38.74	19.46	0.502	0.026	0.9984
(IAI) 0.5	45.68	26.48	0.580	0.022	0.9991
(IAI) 1.0	50.84	30.07	0.591	0.020	0.9998
(IAI) 2.0	57.21	36.35	0.635	0.017	0.9998
(KR) 0	35.99	17.41	0.484	0.028	0.9988
(KR) 2.5	44.31	24.13	0.545	0.023	0.9980
(KR) 5.0	57.70	42.93	0.744	0.017	0.9991
(SG) 0	45.78	18.69	0.408	0.022	0.9962
(SG) 2.5	59.22	34.29	0.579	0.017	0.9973
(SG) 5.0	70.13	64.87	0.925	0.014	0.9976
(SG) 10.0	106.00	139.40	1.315	0.009	0.9992

### 3.3 Molecular docking

**FIGURE 3:**
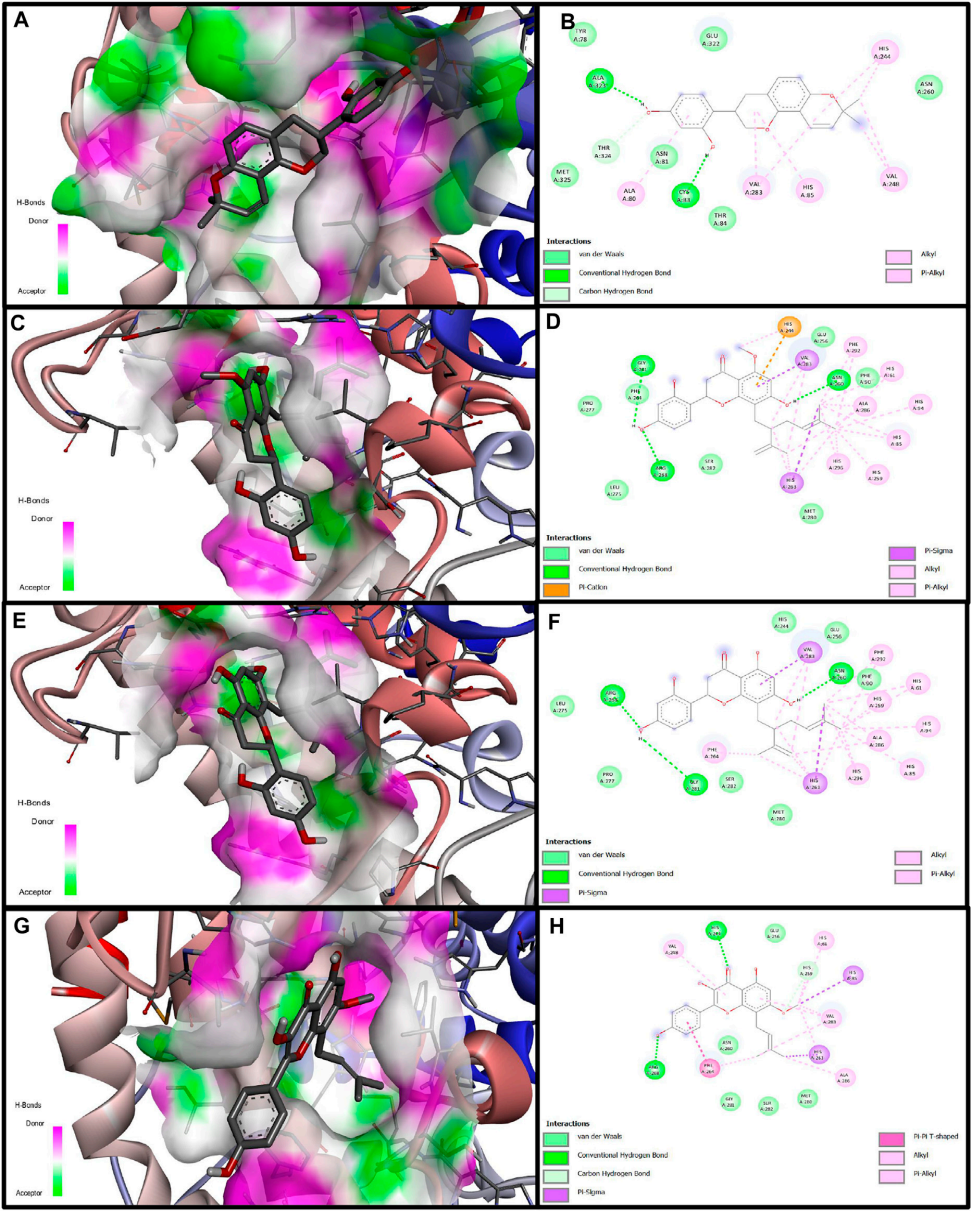
Molecular docking simulation of glabridin, IAI, KR, and SG with tyrosinase. 3D diagram of the docking of compounds with tyrosinase protein molecules, **(A)** Glabridin, **(C)** IAI, **(E)** KR, **(G)** SG. 2D diagram of the interaction between compounds and amino acid residues of tyrosinase proteins, **(B)** Glabridin, **(D)** IAI, **(F)** KR, **(H)** SG.

Computational docking experiments were used to further understand the inhibition mechanism of prenylated flavonoids on tyrosinase. Among the tested flavonoids, SG had the strongest binding affinity with a binding free energy of −8.19 kcal/mol. KR, glabridin, and IAI also exhibited comparable binding affinities with binding free energies of −7.86, −7.04, and −7.03 kcal/mol, respectively. These compounds were able to form two or three hydrogen bonds with the amino acid residues located within this specific pocket. Upon binding, the phenolic hydroxyl group of IAI could create hydrogen bonds with the amino acid residues His244 and Arg268. This was further stabilized by *π-π* and *π-σ* stacking. The primary contact between SG and tyrosinase was hydrogen bonding. Specifically, the phenolic hydroxyl group of SG could create hydrogen bonds with residues of amino acids including Arg268, Gly281, and Asn260, as well as different non-covalent interactions involving van der Waals forces. When bound to amino acid residues such as Gly281, Arg268, and Asn260, the phenolic hydroxyl group of KR could establish hydrogen bonds with them. It could also engage non-covalently with tyrosinase proteins in a variety of ways, such as *π-σ* stacking and *π-*alkyl interaction (Figure 3).

### 3.4 Cytotoxicity in B16F10 cells

**FIGURE 4:**
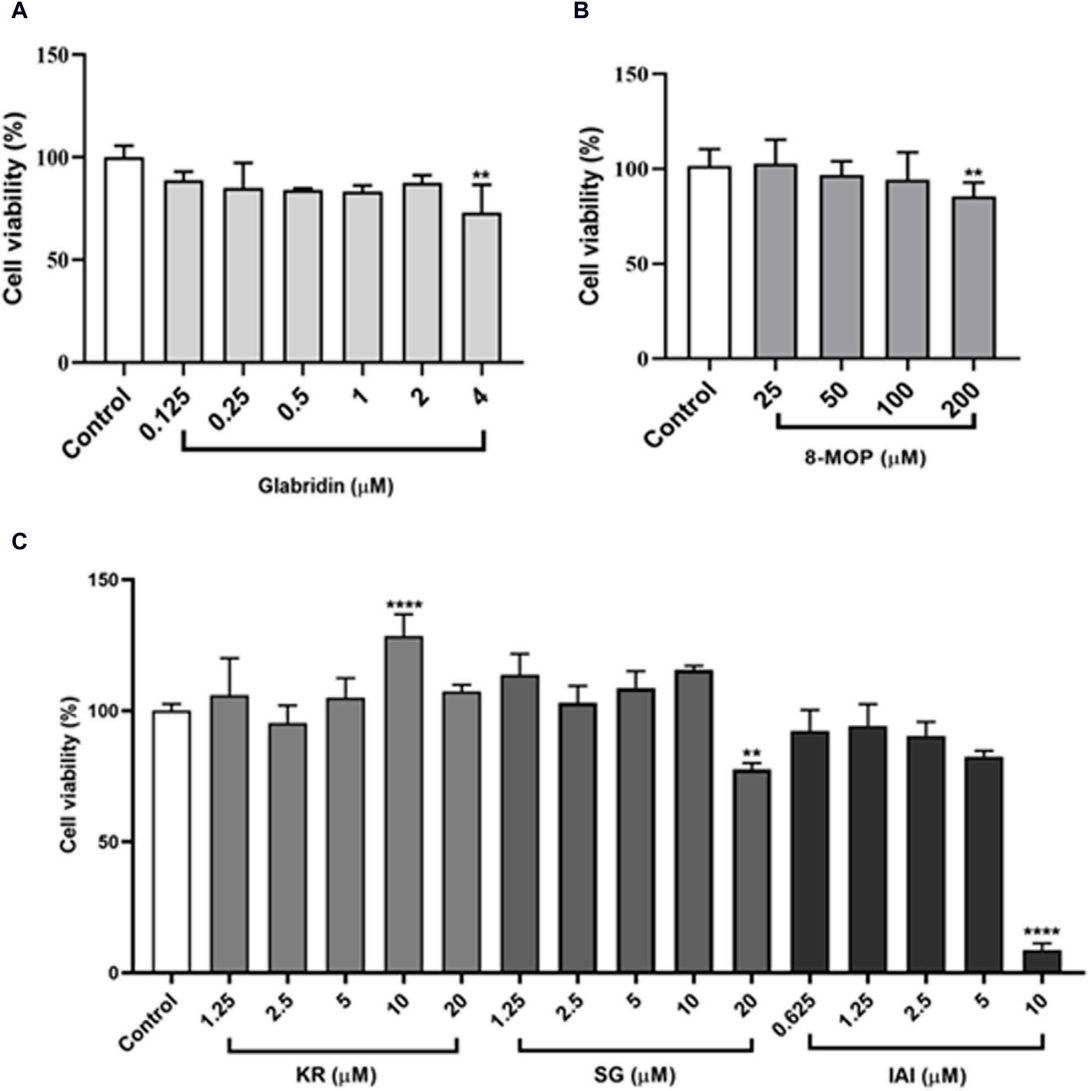
B16F10 cell viability after treatment with various concentrations of test samples. Cells exposed to varying concentrations of test samples for 48 h, cell viability was assessed by MTT assay. **(A)** Glabridin, **(B)** 8-MOP, **(C)** IAI, KR, and SG. The results were expressed as mean ± SD and represent three independent tests. *p < 0.05, **p < 0.01, ***p < 0.001, and ****p < 0.0001 vs. control.

To further evaluate the cellular anti-tyrosinase activity of IAI, KR, and SG, the cytotoxicity of these flavonoids and two positive controls (glabridin and 8-MOP) in B16F10 cells were assessed. As shown in Figure 4, after treatment with different concentrations of IAI (0–5 μM), KR (0–20 μM), and SG (0–10 μM), along with the positive controls glabridin (0–2 μM) and 8-MOP (0–100 μM) for 48 h, the cell viability was maintained above 80%. This indicates these compounds were non-toxic at the indicated concentration range.

### 3.5 Melanin content in B16F10 cells

**FIGURE 5:**
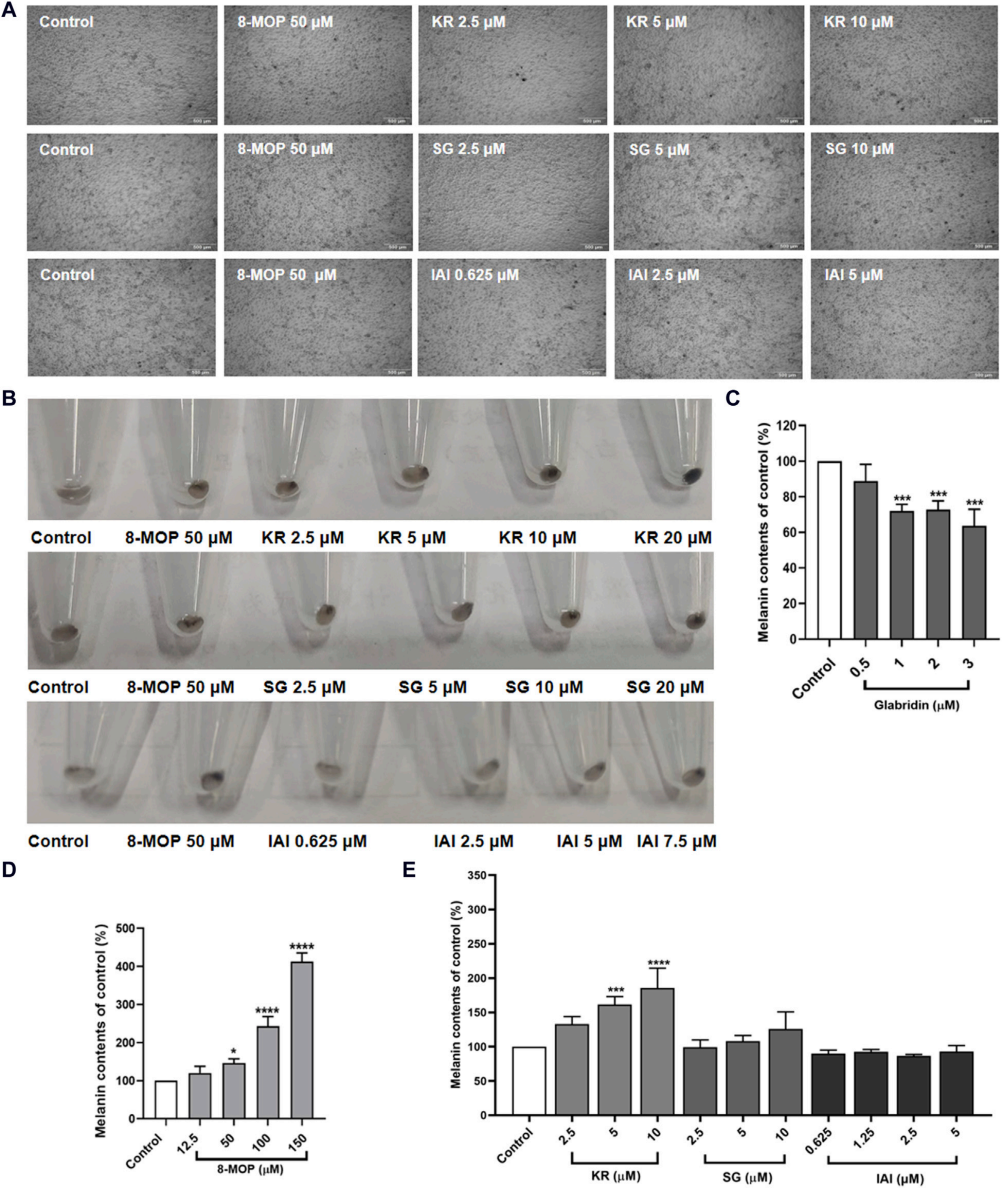
Effects of test samples on melanin production in B16F10 melanoma cells. Cells were treated with test samples at the indicated concentrations for 48 h, glabridin and 8-MOP were used as positive control, respectively. The melanin content was expressed as percentages relative to untreated cells. **(A)** Cell morphology of micrograph, **(B)** Appearance of the recovered cell pellets in test tubes. Contents of extracellular melanin in B16F10 cells treated with samples for 48 h, **(C)** glabridin, **(D)** 8-MOP, **(E)** IAI, KR, and SG. The results were expressed as mean ± SD and represent three independent tests. *p < 0.05, **p < 0.01, ***p < 0.001, and ****p < 0.0001 vs. control.

To identify the effect of IAI, KR, and SG on melanogenesis, B16F10 cells were treated with various concentrations of test samples for 48 h to measure the melanin content. As shown in Figure 5A, cells treated with test compounds were slender and darkened when compared to untreated control cells. The appearance of the collected cell pellets from B16F1 cells was visibly darkened, indicating the accumulation of melanin in the cells (Figure 5B). In addition, the relative melanin content produced in the cells was measured (Figures 5C–E). As expected, glabridin exhibited a strong anti-melanogenic effect and 8-MOP promoted melanin biosynthesis. Among the tested flavonoids, KR increased the intracellular melanin content in B16F10 cells by 61.6% and 85.5% at concentrations of 5 and 10 μM, respectively. SG and IAI did not exert any significant effects on melanin production in B16F10 cells.

### 3.6 Tyrosinase activity in B16F10 cells

**FIGURE 6:**
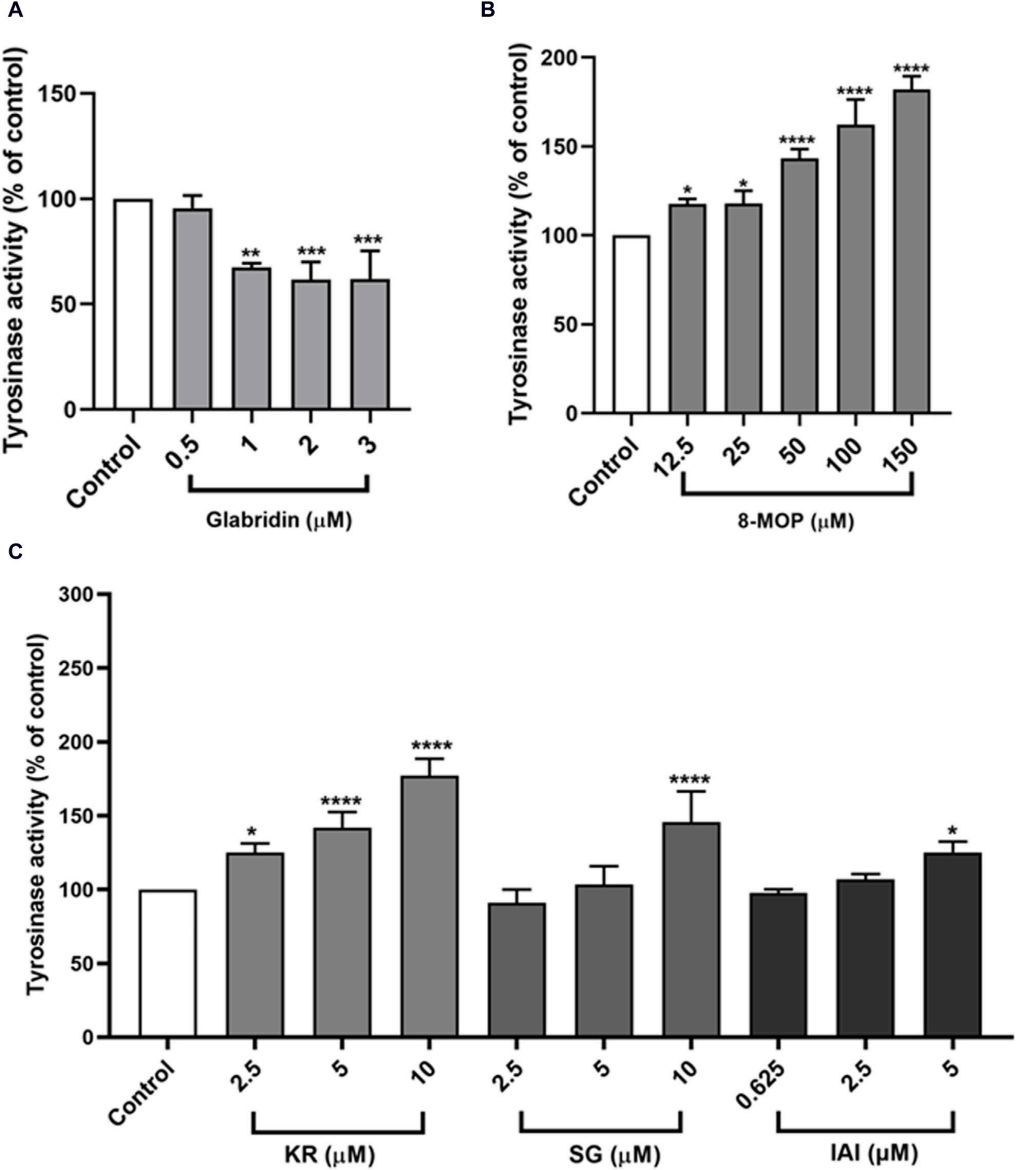
Effects of test samples on tyrosinase activity in B16F10 melanoma cells. Cells were treated with test samples at the indicated concentrations for 48 h, glabridin and 8-MOP were used as positive control, respectively. The tyrosinase activity was expressed as percentages relative to untreated cells. **(A)** Glabridin, **(B)** 8-MOP, **(C)** IAI, KR, and SG. The results were expressed as mean ± SD and represent three independent tests. *p < 0.05, **p < 0.01, ***p < 0.001, and ****p < 0.0001 vs. control.

As tyrosinase is the key enzyme for melanin biosynthesis, we measured the tyrosinase activity in B16F10 cells that were treated with IAI, KR, and SG, along with glabridin and 8-MOP as control groups. As shown in Figures 6A, B, consistent with our findings on melanin production, glabridin inhibited tyrosinase activity and 8-MOP was able to enhance cellular tyrosinase activity. KR enhanced tyrosinase activity in a concentration-dependent manner (increasing 25.1%, 42.1%, and 77.2% enzyme activity by 2.5, 5, and 10 μM). Cells treated with SG and IAI only slightly increased tyrosinase activity at the highest concentration (SG 10 μM and IAI 5 μM) by 45.7% and 25.2%, respectively (Figure 6C).

### 3.7 Effects on melanogenesis signaling pathways

**FIGURE 7:**
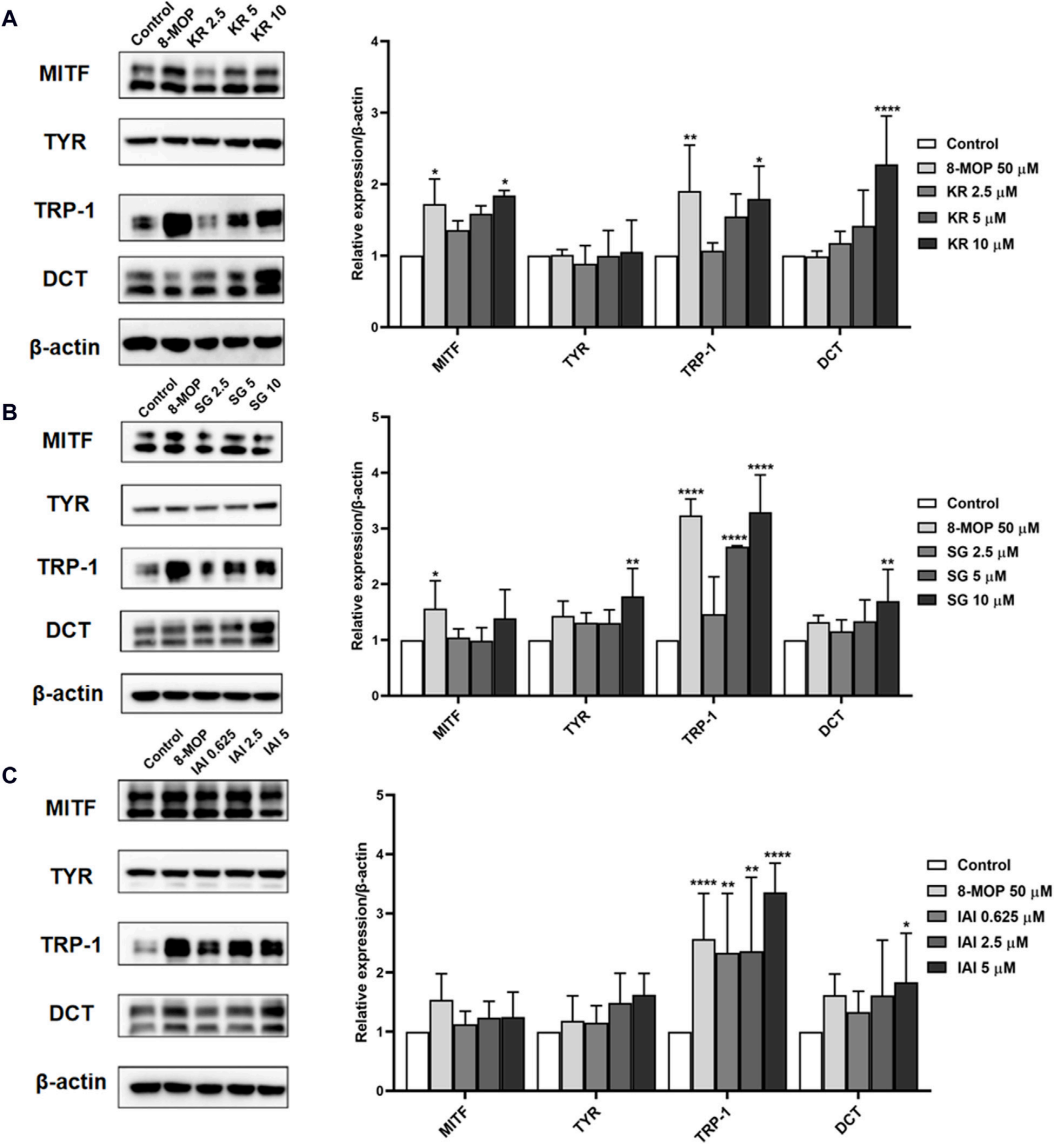
Effects of IAI, KR, and IAI on the protein expression levels of MITF, TYR, TRP-1, and DCT in B16F10 cells. Cells were treated with the indicated concentrations of test samples for 48 h, and 8-MOP was used as a positive control. Protein levels were examined by Western blotting, actin was used as the control, and results were expressed as percentages of untreated cells. **(A)** KR, **(B)** SG, **(C)** IAI. The results were expressed as mean ± SD and represent three independent tests. *p < 0.05, **p < 0.01, ***p < 0.001, and ****p < 0.0001 vs. control.

Melanin synthesis was mainly catalyzed by three melanocyte-specific enzymes, namely, tyrosinase (TYR), tyrosinase-related protein 1 (TRP-1), and dopachrome tautomerase (DCT) (Yin et al., 2018). The expression of these proteins was transcriptionally regulated by the microphthalmia-associated transcription factor (MITF) (Rai et al., 2020). To further explore the mechanisms of IAI, KR, and SG in melanogenesis, the expression of melanogenesis-related proteins such as TRP-1, DCT, MITF, and TYR were evaluated by Western blotting analysis in B16F10 cells (Yin et al., 2018). As shown in Figures 7A–C, the expression of MITF and TRP-1 were increased after the treatment with 8-MOP. After the treatment of IAI at a concentration of 5 μM, the relative expression of TRP-1 and DCT proteins was upregulated by 240.0% and 83.4%, respectively. KR (10 μM) significantly upregulated the relative expression of MITF, TRP-1, and DCT proteins by 84.0%, 79.2%, and 127.8%, respectively. SG (10 μM) increased the expression of TYR, TRP-1, and DCT by 78.4%, 229.3%, and 70.0%, respectively. These findings suggest that IAI, KR, and SG may increase melanogenesis by inducing the expression of relative proteins.

### 3.8 Effects on melanin pigmentation in zebrafish embryos

**FIGURE 8:**
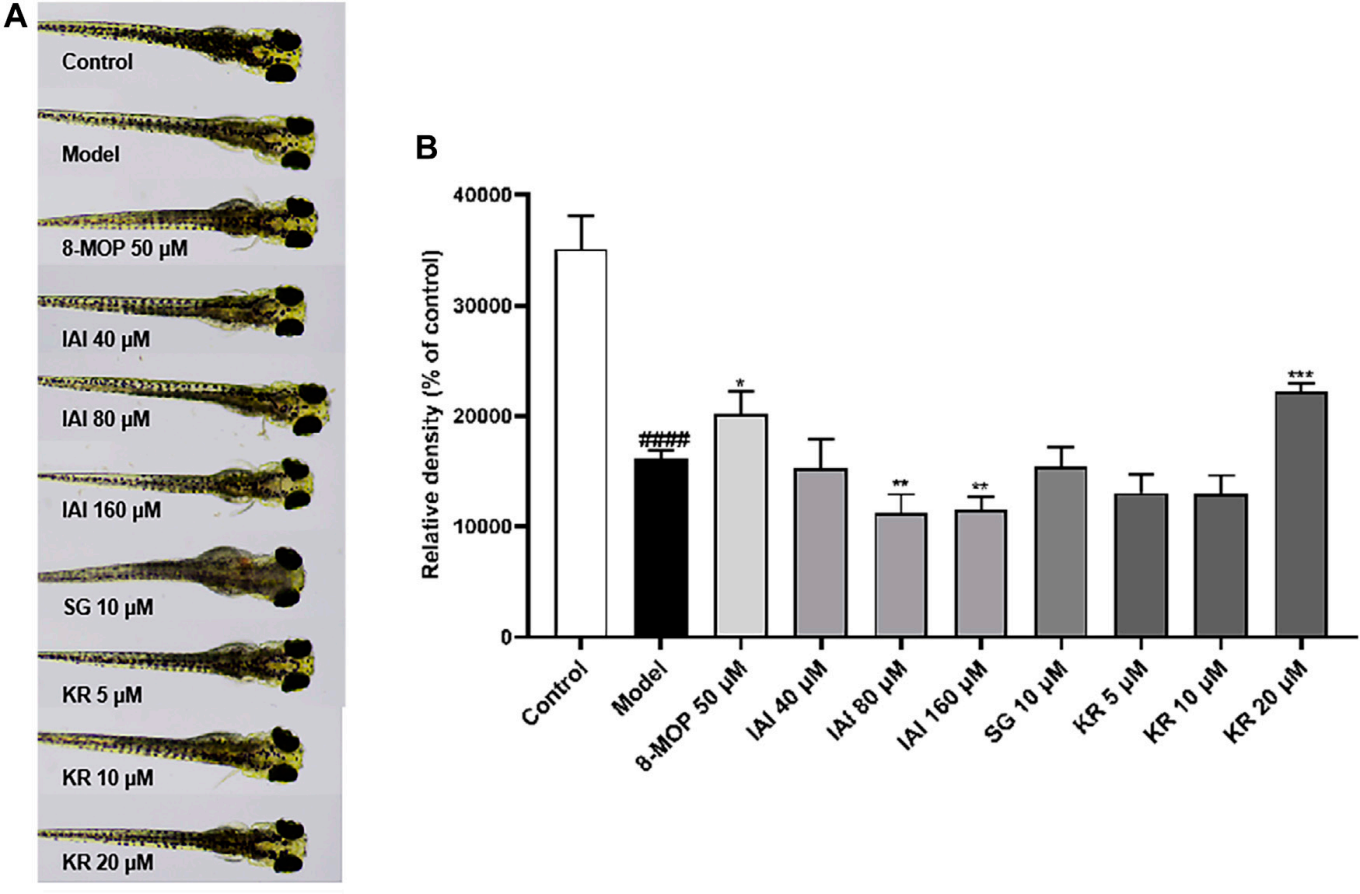
KR upregulates melanin pigmentation in zebrafish larvae. **(A)** The zebrafish larvae (*n* = 40) were collected at 24 hpf and they were treated with PTU (200 μM) for another 24 h. Then, zebrafish larvae were treated with test samples for 48 h (96 hpf), and images were captured at 96 hpf under a microscope, 8-MOP (50 μM) was used as a positive control, and blank control was not treated and was cultured normally. **(B)** Relative density was calculated using Image J software. The results were expressed as mean ± SD and represent three independent tests. ^####^ *p* < 0.0001 vs. control, *p < 0.05, **p < 0.01 and ***p < 0.001 vs. model.

**FIGURE 9:**
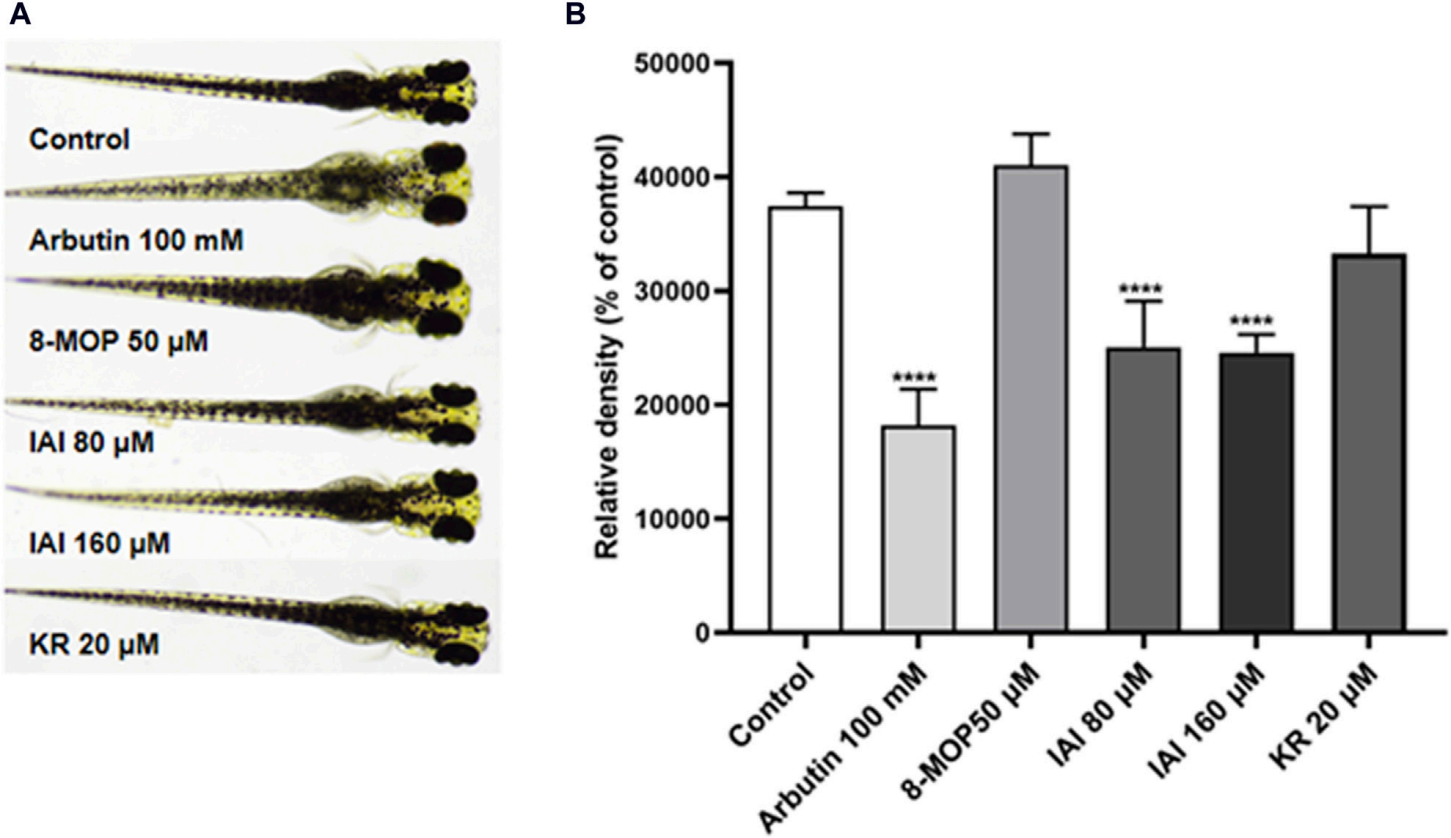
IAI downregulates melanin pigmentation in zebrafish larvae. **(A)** The zebrafish larvae (*n* = 40) were collected at 24 hpf and they were treated with fresh culture media for another 24 h. Then, zebrafish larvae were treated with test samples for 48 h (96 hpf), and images were captured at 96 hpf under a microscope, 8-MOP (50 μM) and arbutin (100 mM) were used as positive controls, blank control was not treated and was cultured normally. **(B)** Relative density was calculated using Image J software. The results were expressed as mean ± SD and represent three independent tests. ****p < 0.0001 vs. control.

To further assess if prenylated flavonoids in *S. flavescens* extract can affect melanogenesis *in vivo*, a zebrafish model was used. The similarity of drug binding regions between human and zebrafish proteins provides a basis for using zebrafish as an *in vivo* suitable model for the evaluation of melanogenesis (Love et al., 2004). First, KR, SG, and IAI were evaluated for their toxicity in zebrafish by the MTC assay. As a result, IAI (40, 80, and 160 μM), KR (5, 10, and 20 μM), and SG (10 μM) were nontoxic to zebrafish and these concentrations were used for subsequent experiments. A small molecule named PTU is a known tyrosinase inhibitor commonly used to block pigmentation and aid visualization of zebrafish development (Escriva et al., 2011). As shown in Figures 8A, B, when compared with the model (PTU) group, the positive controls 8-MOP (50 μM) and KR (20 μM) increased melanin production by 25.0% and 36.9%, respectively. However, SG at 10 μM showed no effect and IAI significantly inhibited melanin production (by 30.8%, and 29.2% at 80 and 160 μM, respectively). In addition, zebrafish without PTU pretreatment were further employed to evaluate the depigmentation effect. As results shown in Figures 9A, B, when compared to the control group, the positive control arbutin (100 mM) reduced pigmentation by 51.4%. IAI also reduced pigmentation by 33.0% and 34.4% at 80 and 160 μM, respectively, whilst 8-MOP and KR did not affect melanin production in this zebrafish model.

## 4 Discussion

Prenylated flavonoids derived from *S. flavescens* are reported as potent tyrosinase inhibitors. However, most of these studies concentrated on the assessment of prenylated flavonoids’ inhibition of tyrosinase (Kim et al., 2003; Son et al., 2003; Ryu et al., 2008). The current study further explored the mechanism of inhibition with enzyme kinetic assays. In addition, several models at the molecular, cellular, and animal levels were used to characterize the modulatory effects of *S. flavescens* prenylated flavonoids on melanin biosynthesis. Notably, SG, kuraridin, and KR had a greater inhibition on tyrosinase activity than the positive control kojic acid (IC_50_ = 6.6 µM, 0.6 µM, and 6.2 µM vs. 20.5 µM, respectively) (Kim et al., 2003). Given that IAI, KR, and SG were the main active compounds in the *S. flavescens* extracts, our findings support the reported overall anti-tyrosinase activity of *S. flavescens*. Moreover, the enzyme kinetics and molecular docking simulation showed that the inhibition type of IAI, KR, and SG on tyrosinase was a mixed type. Among these prenylated flavonoids, KR significantly increased intracellular tyrosinase activity and promoted melanin production in B16F10 cells by activating MITF and its downstream proteins TRP-1 and DCT. This is the first study showing that KR enhances the biosynthesis of melanin. This was further supported by the *in vivo* zebrafish model assessing the effects of IAI, KR, and SG on melanogenesis. With the pretreatment with PTU, IAI substantially inhibited melanin production in zebrafish larvae at nontoxic concentrations. Furthermore, data from the zebrafish whitening model supported the observed inhibition of melanogenesis by IAI. In comparison, KR can stimulate melanin production but SG had no significant effect on melanin formation. These contrasting effects of prenylated flavonoids from *S. flavescens* may partially account for the uses of this medicinal plant for skin lightening and darkening. However, the molecular mechanism of these prenylated flavonoids exerting contrasting effects in melanogenesis is not clear. Further studies are needed to clarify the specific structure-activity relationships (SARs). This is critical as further studies would provide insight into the development of *S. flavescens* based ingredients for cosmeceutical applications or potential medicinal management for vitiligo.

Our study also highlights the importance of using multiple models to evaluate the effects of natural products on melanin biosynthesis and skin pigmentation. This is attributed to bioassays with different models (i.e., enzymatic assays, cell-and animal-based models) that may generate contrasting findings. For instance, IAI showed an anti-tyrosinase effect in the biochemical enzyme assay but was not an inhibitor of tyrosinase in the cellular assay. However, it reduced melanin production in the zebrafish model. This suggests that it is not suitable to evaluate the anti-melanogenic effect solely relying on a single assay. A similar trend was observed where KR demonstrated consistent effects in the cellular and animal models but was inconsistent with the *in vitro* enzyme inhibition assay. This is in agreement with some previously reported studies. Glycyrrhizin acid was reported to stimulate the activity of mushroom tyrosinase but suppress tyrosinase activity in B16F10 cells. In the cell-based model, glycyrrhizin acid was found to increase intracellular melanin content, which was contradictory to the effect in zebrafish showing an anti-melanogenic activity. A human clinical study supported that glycyrrhizin acid indeed can reduce melanin generation (Sun, 2022). Another example is that an Oolong tea extract showed weak inhibition of tyrosinase but reduced melanogenesis in the cellular assay (with B16F10 cells) and *in vivo* guinea pig models (Aoki et al., 2014). Kaempferide, a weak inhibitor of tyrosinase, inhibited melanogenesis in theophylline-stimulated B16 melanoma 4A5 cells (Matsuda et al., 2009), whereas other studies showed that kaempferide dose-dependently enhanced melanin production in both B16F10 cells and C57BL/6 mice (Wang et al., 2017 Wang et al., 2018). Human clinical studies can provide the most relevant and convincing evidence for the evaluation of the modulation of melanogenesis. However, it is not a practical approach to screen natural products as modulators of melanin biosynthesis. Thus, research efforts should be directed to using rigorous models with data from molecular assays to provide profound information.

In summary, we evaluated the anti-tyrosinase activity and melanogenesis effects of four prenylated flavonoids from *S. flavescens* extracts. Among these flavonoids, IAI showed promising tyrosinase inhibitory activity and anti-melanogenic effect in the zebrafish model. KR promoted melanin production in B16F10 cells and the zebrafish model. Although IAI and KR from *S. flavescens* may have contrasting effects on melanogenesis, they might both be promising candidates as modulators for melanin production with different cosmeceutical or medicinal purposes.

## Data Availability

The original contributions presented in the study are included in the article/Supplementary Material, further inquiries can be directed to the corresponding authors.

## References

[B1] Ahmed M. B. Islam S. U. Lee Y. S. (2021). PRP4 promotes skin cancer by inhibiting production of melanin, blocking influx of extracellular calcium, and remodeling cell actin cytoskeleton. Int. J. Mol. Sci. 22 (13), 6992. 10.3390/ijms22136992 34209674 PMC8268783

[B2] Aoki Y. Tanigawa T. Abe H. Fujiwara Y. (2014). Melanogenesis inhibition by an oolong tea extract in B16 mouse melanoma cells and UV-induced skin pigmentation in brownish Guinea pigs. Biosci. Biotechnol. Biochem. 71 (8), 1879–1885. 10.1271/bbb.70099 17690471

[B3] Butt A. R. S. Abbasi M. A. Aziz ur R. Siddiqui S. Z. Raza H. Hassan M. (2019). Synthesis and structure-activity relationship of tyrosinase inhibiting novel bi-heterocyclic acetamides: mechanistic insights through enzyme inhibition, kinetics and computational studies. Bioorg. Chem. 86, 459–472. 10.1016/j.bioorg.2019.01.036 30772647

[B4] Cai Y. Cai Q. Liu Z. Huang P. Han H. (2011). Chinese medicine preparation for treating vitiligo. Hebei China Natl. Intellect. Prop. Adm. C. N. Pat. No 102, 228–522.

[B5] Choi H. Yoon J.-H. Youn K. Jun M. (2022). Decursin prevents melanogenesis by suppressing MITF expression through the regulation of PKA/CREB, MAPKs, and PI3K/Akt/GSK-3β cascades. Biomed. Pharmacother. 147, 112651. 10.1016/j.biopha.2022.112651 35063859

[B6] Del Marmol V. Beermann F. (1999). Tyrosinase and related proteins in mammalian pigmentation. FEBS Lett. 381 (3), 165–168. 10.1016/0014-5793(96)00109-3 8601447

[B7] Ding Q. Luo L. Yu L. Huang S.-l. Wang X.-q. Zhang B. (2021). The critical role of glutathione redox homeostasis towards oxidation in ermanin-induced melanogenesis. Free Radic. Biol. Med. 176, 392–405. 10.1016/j.freeradbiomed.2021.09.017 34560247

[B8] D’Mello S. Finlay G. Baguley B. Askarian-Amiri M. (2016). Signaling pathways in melanogenesis. Int. J. Mol. Sci. 17 (7), 1144. 10.3390/ijms17071144 27428965 PMC4964517

[B9] Escriva H. Bohnsack B. L. Gallina D. Kahana A. (2011). Phenothiourea sensitizes zebrafish cranial neural crest and extraocular muscle development to changes in retinoic acid and IGF signaling. PLoS ONE 6 (8), e22991. 10.1371/journal.pone.0022991 21886774 PMC3158757

[B10] Grimes P. Nordlund J. J. Pandya A. G. Taylor S. Rendon M. Ortonne J.-P. (2006). Increasing our understanding of pigmentary disorders. J. Am. Acad. Dermatol. 54 (5), S255–S261. 10.1016/j.jaad.2005.12.042 16631966

[B11] Han H. J. Park S. K. Kang J. Y. Kim J. M. Yoo S. K. Heo H. J. (2020). Anti-melanogenic effect of ethanolic extract of sorghum bicolor on IBMX-induced melanogenesis in B16/F10 melanoma cells. Nutrients 12 (3), 832. 10.3390/nu12030832 32245029 PMC7146600

[B12] He X. Fang J. Huang L. Wang J. Huang X. (2015). *Sophora flavescens* Ait.: traditional usage, phytochemistry and pharmacology of an important traditional Chinese medicine. J. Ethnopharmacol. 172, 10–29. 10.1016/j.jep.2015.06.010 26087234

[B13] Heriniaina R. M. Dong J. Kalavagunta P. K. Wu H.-L. Yan D.-S. Shang J. (2018). Effects of six compounds with different chemical structures on melanogenesis. Chin. J. Nat. Med. 16 (10), 766–773. 10.1016/s1875-5364(18)30116-x 30322610

[B14] Hyun S. K. Lee W.-H. Jeong D. M. Kim Y. Choi J. S. (2008). Inhibitory effects of kurarinol, kuraridinol, and trifolirhizin from *Sophora flavescens* on tyrosinase and melanin synthesis. Biol. Pharm. Bull. 31 (1), 154–158. 10.1248/bpb.31.154 18175961

[B15] Jang D. K. Pham C. H. Lee I. S. Jung S.-H. Jeong J. H. Shin H.-S. (2020). Anti-melanogenesis activity of 6-o-isobutyrylbritannilactone from *Inula britannica* on B16F10 melanocytes and *in vivo* zebrafish models. Molecules 25 (17), 3887. 10.3390/molecules25173887 32858952 PMC7504228

[B16] Karunarathne W. A. H. M. Molagoda I. M. N. Kim M. S. Choi Y. H. Oren M. Park E. K. (2019). Flumequine-mediated upregulation of p38 MAPK and JNK results in melanogenesis in B16F10 cells and zebrafish larvae. Biomolecules 9 (10), 596. 10.3390/biom9100596 31614510 PMC6843389

[B17] Kim C. Noh S. Park Y. Kang D. Chun P. Chung H. (2018a). A potent tyrosinase inhibitor, (E)-3-(2,4-Dihydroxyphenyl)-1-(thiophen-2-yl)prop-2-en-1-one, with anti-melanogenesis properties in α-MSH and IBMX-induced B16F10 melanoma cells. Molecules 23 (10), 2725. 10.3390/molecules23102725 30360412 PMC6222382

[B18] Kim J. H. Cho I. S. So Y. K. Kim H.-H. Kim Y. H. (2018b). Kushenol A and 8-prenylkaempferol, tyrosinase inhibitors, derived from *Sophora flavescens* . J. Enzyme Inhib. Med. Chem. 33 (1), 1048–1054. 10.1080/14756366.2018.1477776 29873272 PMC6009905

[B19] Kim S. J. Son K. H. Chang H. W. Kang S. S. Kim H. P. (2003). Tyrosinase inhibitory prenylated flavonoids from *Sophora flavescens* . Biol. Pharm. Bull. 26 (9), 1348–1350. 10.1248/bpb.26.1348 12951485

[B20] Lajis A. F. B. Ariff A. B. (2019). Discovery of new depigmenting compounds and their efficacy to treat hyperpigmentation: evidence from *in vitro* study. J. Cosmet. Dermatol. 18 (3), 703–727. 10.1111/jocd.12900 30866156

[B21] Li D.-L. Zheng X. Chen Y.-C. Jiang S. Zhang Y. Zhang W.-M. (2015). Terpenoid composition and the anticancer activity of *Acanthopanax trifoliatus* . Arch. Pharm. Res. 39 (1), 51–58. 10.1007/s12272-015-0655-y 26345267

[B22] Love D. R. Pichler F. B. Dodd A. Copp B. R. Greenwood D. R. (2004). Technology for high-throughput screens: the present and future using zebrafish. Curr. Opin. Biotechnol. 15 (6), 564–571. 10.1016/j.copbio.2004.09.004 15560983

[B23] Maack A. Pegard A. (2016). *Populus nigra* (Salicaceae) absolute rich in phenolic acids, phenylpropanoids and flavonoids as a new potent tyrosinase inhibitor. Fitoterapia 111, 95–101. 10.1016/j.fitote.2016.04.001 27091790

[B24] Matsuda H. Nakashima S. Oda Y. Nakamura S. Yoshikawa M. (2009). Melanogenesis inhibitors from the rhizomes of *Alpinia officinarum* in B16 melanoma cells. Bioorg. Med. Chem. 17 (16), 6048–6053. 10.1016/j.bmc.2009.06.057 19615910

[B25] Mort R. L. Jackson I. J. Patton E. E. (2015). The melanocyte lineage in development and disease. Development 142 (4), 620–632. 10.1242/dev.106567 25670789 PMC4325379

[B26] Niu C. Aisa H. A. (2017). Upregulation of melanogenesis and tyrosinase activity: potential agents for vitiligo. Molecules 22 (8), 1303. 10.3390/molecules22081303 28777326 PMC6152334

[B27] Park S. Y. Jin M. L. Kim Y. H. Kim Y. Lee S.-J. (2011). Aromatic-turmerone inhibits α-MSH and IBMX-induced melanogenesis by inactivating CREB and MITF signaling pathways. Arch. Dermatol. Res. 303 (10), 737–744. 10.1007/s00403-011-1155-7 21660443

[B28] Pillaiyar T. Manickam M. Namasivayam V. (2017). Skin whitening agents: medicinal chemistry perspective of tyrosinase inhibitors. J. Enzyme Inhib. Med. Chem. 32 (1), 403–425. 10.1080/14756366.2016.1256882 28097901 PMC6010116

[B29] Rai A. Chatterjee B. Bhowmick S. Sagar S. Roy S. S. (2020). Beclin 1 controls pigmentation by changing the nuclear localization of melanogenic factor MITF. Biochem. Biophys. Res. Commun. 528 (4), 719–725. 10.1016/j.bbrc.2020.05.118 32513537

[B30] Ryu Y. B. Westwood I. M. Kang N. S. Kim H. Y. Kim J. H. Moon Y. H. (2008). Kurarinol, tyrosinase inhibitor isolated from the root of *Sophora flavescens* . Phytomed 15 (8), 612–618. 10.1016/j.phymed.2007.09.022 17951038

[B31] Shin D. H. Cha Y. J. Joe G. J. Yang K. E. Jang I.-S. Kim B. H. (2013). Whitening effect of *Sophora flavescensextract* . Pharm. Biol. 51 (11), 1467–1476. 10.3109/13880209.2013.799708 24106757

[B32] Slominski A. Tobin D. J. Shibahara S. Wortsman J. (2004). Melanin pigmentation in mammalian skin and its hormonal regulation. Physiol. Rev. 84 (4), 1155–1228. 10.1152/physrev.00044.2003 15383650

[B33] Son J. K. Park J. S. Kim J. A. Kim Y. Chung S. R. Lee S. H. (2003). Prenylated flavonoids from the roots of *Sophora flavescens* with tyrosinase inhibitory activity. Planta Med. 69 (6), 559–561. 10.1055/s-2003-40643 12865979

[B34] Sun R. Bao G. (2008). Chinese medicinal tincture for treating facial vitiligo. Zhejiang: China National Intellectual Property Administration. C. N. Patent No 101.

[B35] Sun T. (2022). Efficacy evaluation and comparative study of four whitening efficacy evaluation models on several whitening ingredients. [Guangdong]: Guangdong University of Technology. [MA thesis].

[B36] Wang J. Wang X. Tang Y. Zhang B. (2018). The network pharmacological mechanisms of four anti-vitiligo Uyghur medicines based on Phlegmatic temperament theory. China J. Chin. Materia Medica 43 (09), 1780–1788. 10.19540/j.cnki.cjcmm.2018.0061 29902886

[B37] Wang J. Y. Chen H. Wang Y. Y. Wang X. Q. Chen H. Y. Zhang M. (2017). Network pharmacological mechanisms of *Vernonia anthelmintica (L.)* in the treatment of vitiligo: isorhamnetin induction of melanogenesis via up-regulation of melanin-biosynthetic genes. BMC Syst. Biol. 11 (1), 103. 10.1186/s12918-017-0486-1 29145845 PMC5691595

[B38] Xu W. Li J. Li D. Tan J. Ma H. Mu Y. (2021). Chemical characterization, antiproliferative and antifungal activities of *Clinacanthus nutans* . Fitoterapia 155, 105061. 10.1016/j.fitote.2021.105061 34673146

[B39] Yasumoto K.-I. Yokoyama K. Shibata K. Tomita Y. Shibahara S. (1994). Microphthalmia-associated transcription factor as a regulator for melanocyte-specific transcription of the human tyrosinase gene. Mol. Cell. Biol. 14 (12), 8058–8070. 10.1128/mcb.14.12.8058 7969144 PMC359344

[B40] Yasumoto K.-I. Yokoyama K. Takahashi K. Tomita Y. Shibahara S. (1997). Functional analysis of microphthalmia-associated transcription factor in pigment cell-specific transcription of the human tyrosinase family Genes. J. Biol. Chem. 272 (1), 503–509. 10.1074/jbc.272.1.503 8995290

[B41] Yin L. Pang G. Niu C. Habasi M. Dou J. Aisa H. (2018). A novel psoralen derivative-MPFC enhances melanogenesis via activation of p38 MAPK and PKA signaling pathways in B16 cells. Int. J. Mol. Med. 41 (6), 3727–3735. 10.3892/ijmm.2018.3529 29512683

[B42] Yoshino M. Murakami K. (2009). A graphical method for determining inhibition constants. J. Enzyme Inhib. Med. Chem. 24 (6), 1288–1290. 10.3109/14756360902829766 19912063

[B43] Yoshioka Y. Samukawa Y. Yamashita Y. Ashida H. (2020). 4-Hydroxyderricin and xanthoangelol isolated from Angelica keiskeiprevent dexamethasone-induced muscle loss. Food Funct. 11 (6), 5498–5512. 10.1039/d0fo00720j 32510085

[B44] Zhang J. Wu X. Zhong B. Liao Q. Wang X. Xie Y. (2023). Review on the diverse biological effects of glabridin. Drug Des. Devel Ther. 17, 15–37. 10.2147/DDDT.S385981 PMC9840373 36647530

[B45] Zhang Q. Zhang Q. (2020). Method for preparation of traditional Chinese medicine for treating vitiligo. Shanxi China Natl. Intellect. Prop. Adm. C. N. Pat. No 111 (494).

[B46] Zhang Y. Sun L. Lai X. Peng X. Wen S. Zhang Z. (2021). Gastroprotective effects of extract of *Jasminum grandiflorum* L. flower in HCl/EtOH-induced gastric mucosal ulceration mice. Biomed. Pharmacother. 144, 112268. 10.1016/j.biopha.2021.112268 34634558

[B47] Zhou X. Fouda S. Zeng X.-Y. Li D. Zhang K. Xu J. (2019). Characterization of the therapeutic profile of albiflorin for the metabolic syndrome. Front. Pharmacol. 10, 1151. 10.3389/fphar.2019.01151 31680948 PMC6797612

